# Clashing in Murky Waters: On Amphibian Mosquito Suppression

**DOI:** 10.1002/ece3.73127

**Published:** 2026-03-07

**Authors:** S. P. Boerlijst, A. Ummels, A. M. Spitzen‐van der Sluijs, J. Spitzen, R. W. Bouman, E. Boelee, P. M. van Bodegom, M. Schrama

**Affiliations:** ^1^ Institute for Environmental Sciences Leiden, Department of Environmental Biology University of Leiden Leiden the Netherlands; ^2^ Deltares, Division of Inland Water Systems Delft the Netherlands; ^3^ Ministry of Defence Den Haag the Netherlands; ^4^ Reptile, Amphibian and Fish Conservation the Netherlands Nijmegen the Netherlands; ^5^ Centre for Monitoring of Vectors, Netherlands Food and Consumer Product Safety Authority Ministry of Agriculture, Nature and Food Quality Wageningen the Netherlands; ^6^ Laboratory of Entomology Wageningen University and Research Wageningen the Netherlands; ^7^ Hortus Botanicus Leiden Leiden the Netherlands; ^8^ Naturalis Biodiversity Center Leiden the Netherlands; ^9^ Institute of Biology Leiden Leiden University Leiden the Netherlands

**Keywords:** amphibian, biological control, control agent, *Culex pipiens*, *Lissotriton vulgaris*, *Pelophylax* kl. *esculentus*, urban green spaces

## Abstract

Mosquito‐borne diseases are on the rise globally due to the shifting distribution of key disease vector species. Due to increased global trade and travel and increasing temperatures and changing precipitation patterns, the spread of mosquitoes and the increasing burden of their accompanying diseases like malaria, yellow fever, and dengue persist. Seeking sustainable control measures, there's growing interest in natural control, particularly through predators like amphibians, a globally threatened vertebrate group. However, the effectiveness of different natural predators and their role in an ecological context remains poorly understood. This study compares the predator efficiency of common European amphibian species to common aquatic insect mosquito predators. Focusing on the cosmopolitan mosquito *Culex pipiens* s.l.*,* known for transmitting pathogens like West Nile virus, we assessed predator rates, sex‐specific efficiency in amphibians, and the impact of predator presence on mosquito oviposition behavior. Amphibians proved to be more effective mosquito predators than aquatic insects, consuming up to 4–8 times as many larvae per individual compared to the aquatic insects. No difference was detected between the two amphibian species, nor their sexes or the levels of eutrophication. Predator cues deterred oviposition behavior across the entire experimental setup, thus suggesting the ability of mosquitoes to react to a (purported) landscape of fear. The combined effect of deterring egg laying and lowering mosquito survival highlights the potential of natural predation, and particularly that of amphibian species as natural larval control agents, thus emphasizing the importance of conserving these threatened species and facilitating them in urban and rural environments.

## Graphical abstract

This study compares the predator efficiency of common European amphibian species to common invertebrate mosquito predators. Focusing on the cosmopolitan mosquito *Culex pipiens*, known for transmitting pathogens like West Nile virus, we assessed predator rates, sex‐specific efficiency in amphibians, and the impact of predator presence on mosquito oviposition behavior. The combined effect of deterring egg laying and lowering mosquito survival highlight the potential of natural predation, and particularly those of amphibian species as natural larval control agents, once highlighting the importance of conserving these—globally threatened—species and facilitating them in urban and rural environments.

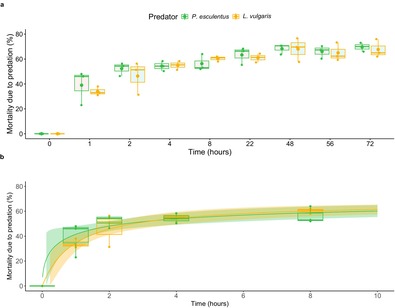

## 1 Introduction

In recent decades, distributions of several mosquito species that are key vectors of diseases have been shifting rapidly towards the global north (Farooq et al. 2025) and towards anthropogenic disturbances (Neiderud 2015; Steiger et al. 2012). This has contributed to an expansion of pathogen distributions and associated increases in disease risk globally (Colón‐González et al. 2021; Kraemer et al. 2015; Roche et al. 2015). Despite extensive mosquito control efforts by governments and healthcare sectors in the global south, and increasing efforts in the global north (Medlock et al. 2012), mosquito‐borne diseases are causing a rising burden of disease (Ferguson 2018). This includes an expansion of the *Culex*‐transmitted West Nile virus to North America and Europe over recent decades, with infections recently reaching Germany and the Netherlands (Brüssow and Figuerola 2025; Hadfield et al. 2019).

Control of larval mosquitoes, most effective for the immature stages (Killeen et al. 2002), is strongly geared towards chemical interventions including insect growth regulators, microbial toxins (Bti), organophosphates, neonicotinoids and pyrethroids. In general, these measures are expensive due to the necessity of regular use, and their long‐term effectiveness has been widely questioned due to increasing resistances (Hamdan et al. 2005; Nazni et al. 2005; Paris et al. 2011). Furthermore, direct or indirect effects on non‐target organisms pose additional threats for biodiversity loss (Antwi and Reddy 2015; Lawler 2017; Moura and Souza‐Santos 2020; Thompson et al. 2020). As this decline in biodiversity may cause alleviation in predation pressure, it could potentially result in widespread increases in mosquito populations (Perrin et al. 2023). Promoting ecologically healthy systems, that is, ecosystems with an abundance of natural predators, as part of a One Health approach, has therefore gained increasing support. With this came an increasing interest in biocontrol agents like plant‐borne mosquitocides, entomopathogenic fungi and particularly mosquito predators (Benelli et al. 2016), but more information is needed on which natural predators effectively suppress mosquito populations (in ephemeral water bodies), or how (neighboring) habitats could be adapted to facilitate them (Carlson et al. 2004).

The importance of natural control agents, and how their effectiveness differs in complex systems that include (interspecific) competition, remains poorly understood (Shaalan and Canyon 2009). Even though predator–prey relationships are generally well‐studied, information in the context of mosquitoes is relatively scarce, especially when considering natural conditions. This is particularly worrisome as large‐scale decreases in potential predator populations were recently reported (Eisenhauer et al. 2023; Kehoe et al. 2021). Impacts on public health are therefore hard to predict, as loss of natural control agents may necessitate alternative forms of mosquito control.

Understanding the role of different mosquito predators requires considering species specificity as a key factor. In general, predator effectiveness—that is, kill rate or consumed prey over time—is a species‐specific interaction, as anti‐predator behavior differs across species and may enhance or impair kill rates (Ohba and Ushio 2015; Sih 1986). Meta analyses of predation efficiency exist for groups such as dragonflies (Odonata:Anisoptera) and damselflies (Odonata:Zygoptera) (Priyadarshana and Slade 2023) and mayflies (Ephemeroptera) (Dasrat and Maharaj 2021), which prey on mosquitoes during their aquatic life stages (Dasrat and Maharaj 2021; Priyadarshana and Slade 2023). However, these analyses are limited to specific groups of (often aquatic insect) predators (Benelli 2015; Lacey and Orr 1994; McDonald and Buchanan 1981), while amphibian predators have been hypothesized to be far more efficient predators (DuRant and Hopkins 2008).

While information on predation rates of a subset of aquatic insect predator taxa are available, amphibians are a poorly understood mosquito predator group (Benelli 2015), particularly their importance in relation to aquatic insects. Incidentally they have been suggested to be effective natural control agents, because, like mosquitoes, they often depend on ephemeral water bodies like wetlands (Brodman and Dorton 2006; DuRant and Hopkins 2008) and incidentally artificial habitats like storm drains (Rey et al. 2006; Holzer 2014). This is in marked contrast to fish such as mosquitofish (Chandra et al. 2008), well‐known predators of mosquitoes that require permanent water and have been introduced numerous times (Chandra et al. 2008). Aside from lowering mosquito survival, there may be other direct or indirect inter‐specific interactions that adversely impact the development of mosquito larvae in shared habitats (Alto et al. 2012; Fischer et al. 2012; Meadows et al. 2017; Russell et al. 2022). Tadpoles, for instance, may prey on mosquito eggs (Weterings 2015), consume similar food resources as mosquito larvae, predate on mosquito larvae during their later developmental stages and affect mosquito habitat choice (Spielman and Sullivan 1974). This may point at a potential effect of competition for food resources between the mosquito larvae and tadpoles, which was previously found in a study with crustacean (Cladocera) competitors, where recolonization by mosquitoes was prevented (Kroeger et al. 2013). As such, predator species with herbivore immature stages like frogs, may deter, limit and even prevent mosquitoes from establishing. However, a broad scale inventory of predatorial capacity including amphibian taxa is hitherto only available as meta‐analysis (Russell et al. 2022) and therefore it is not known how predation by aquatic insect and amphibian predators compare across ecological contexts.

As such, we aimed to compare the predator effectiveness of two common European amphibians—the smooth newt (Caudata: *Lissotriton vulgaris* ) and the edible frog (Anura: *Pelophylax* kl. *esculentus*)—against two common European aquatic insect mosquito predators found in ephemeral habitats (including urban green/blue): the two‐spot water beetle (Coleoptera: *Agabus bipustulatus* ; Culler and Lamp 2009) and backswimmer (Hemiptera: *Notonecta glauca* ; Saha et al. 2010) across a range of ecologically realistic conditions. These amphibian species were previously classified under the genera *Triturus* and *Rana* respectively, which include widely distributed species across Asia and Europe. We focused on common predator species that can colonize ephemeral water bodies by, that is, their ability to breathe air and migrate over land, like true bugs (Fischer et al. 2012), beetles (Lundkvist et al. 2003) and amphibians (Brodman and Dorton 2006), the latter of which may disperse several hundred meters (Müllner 2001; Peter 2001; Schmidt et al. 2006). Similarly, we focused on larger predator species as they are less temperature dependent (Van Der Have and De Jong 1996), and as food intake increases with size of the animal (DuRant and Hopkins 2008; Jennings et al. 2002; Robinson et al. 1983). Both *L. vulgaris* and *P. esculentus* are known to feed while in and under water (Anamaria et al. 2011; Blommers‐Schlösser 1992; Covaciu‐Marcov 2010; Sas et al. 2007, 2009; Tyler 1958) and Nematocera larvae have been incidentally described as a food source (Anamaria et al. 2011; Covaciu‐Marcov 2010; Sas et al. 2007). Only adult frogs and newts were used during these experiments. This is necessary for frogs specifically as development of the mouthparts allow for carnivorous diet only during the short window between Gosner stages 42 and 46, representing the final larval stages before transformation marked by the appearance of legs and disappearance of the tail (Gosner 1960; Johansson et al. 2010).

Experiments were conducted to assess (i) the predator effectiveness of selected aquatic insect and amphibian species across different levels of eutrophication, (ii) whether there is a sex specificity of predator effectiveness of selected amphibian species, and (iii) the effect of predator presence on mosquito oviposition behavior.

To address these aims, we conducted a series of experiments determining and comparing the predation rate on *Culex pipiens* . We focus on *Culex pipiens* s.l., a common and cosmopolitan species with a wide tolerance to temperature (7°C–35°C; Loetti et al. 2011) and eutrophication (0–100 mg N‐total; Boerlijst et al. 2023), known to occupy almost every type of water body (Becker et al. 2010). *Culex* as a genus represents the predominant vectors of West Nile virus, Usutu, Japanese encephalitis, Avian malaria and Dirofilariasis among other pathogens, of which the *Culex pipiens* species group is the most widespread (Harbach 2012). Due to its locally high abundance, wide distribution range and its aptitude for transmission of a variety of pathogens, it is an important vector and nuisance species. *Culex pipiens* thrives in small aquatic systems (Buxton et al. 2020), especially under hypertrophic and even anoxic conditions (Boerlijst et al. 2023).

Predatory behavior on the mosquito genera *Aedes* and *Anopheles* was validated for *L. vulgaris* and *Pelophylax kl. esculentus* to determine whether our results could translate to other mosquito taxa.

## 2 Methods

### 2.1 Experimental Setup

The experiments consist of (i) a comparison in predator effectiveness of *L. vulgaris* , *A. bipustulatus* and *N. glauca* , (ii) an assessment of *L. vulgaris* predator effectiveness across different eutrophic levels, (iii) a large‐scale comparison of sex‐specific predator effectiveness of *L. vulgaris* and *P. esculentus* , (iv) an assessment of amphibian presence on mosquito oviposition, and (v) a comparison of amphibian predator effectiveness on *Aedes* and *Anopheles* larvae. All experiments were performed in a full factorial setup under outdoor conditions (Table 1).

**TABLE 1:** Mesocosm set‐up for each of the experiments. Ratios of replicates are represented per treatment level.

Experiment	Mesocosm	Replicates (treatment/control)	Density of mosquito larvae	Treatments
1	16 L white polypropylene	3/3	50	Predator ( *L. vulgaris* , *A. bipustulatus* , *N. glauca* , control)
2	16 L white polypropylene	8/4	50	Predator ( *L. vulgaris* , control), eutrophication (4 mg/L, 8 mg/L)
3	48 L black polypropylene	11/14	50	Predator ( *L. vulgaris* , *P. esculentus* , control), Sex (male, female)
4	48 L black polypropylene	11/14	50	Predator ( *L. vulgaris* , *P. esculentus* , control), Sex (male, female)
5	20 L aquarium	8/0^a^	3	Predator ( *L. vulgaris* , *P. esculentus* )

^a^ No controls were included as this experiment concerns direct observations of hunting behavior.

#### 2.1.1 Pre‐Experimental Conditions

For each experiment, we used a set of similar pre‐experimental conditions. A series of mesocosms (Table 1) was placed in a randomized full‐factorial grid. The mesocosms are representative of the artificial containers that *Cx. pipiens* is known to colonize (Koenraadt and Harrington 2008; Boerlijst et al. 2023). Each mesocosm was filled with dechlorinated tap water and a standardized community of algae and bacteria, collected with a plankton net (250 μm with a 53 μm collector) from the lake next to the Living Lab field station (Leiden, The Netherlands), where all selected predator species naturally occur. The 250 μm net served to remove algal mats prior to filtering the phytoplankton on the 53 μm collector. The filtered algae and bacteria obtained were divided equally across all mesocosms so that 1 liter of water in the set‐up contained as much microbes as a liter of ditch water (Dellar et al. 2022). Eutrophic levels representative of Dutch ditches (4–10 mg/L N‐total; Loeb and Verdonschot 2008) were created, using cow manure pellets (2,4% N; 1,5% P_2_O_5_; 3,1% K_2_O) (Boerlijst et al. 2023). As such, 4 and 8 mg N‐total was used for experiment 2 and 4 mg N‐total for all other experiments. After a day of acclimation, the contents of the mesocosms were stirred and strained through a 300 μm sieve to remove any large particulate matter. The mesocosms were then covered with a 0.1 mm mesh to prevent natural colonization by mosquitoes and predators. The bacterial community was thereafter left to acclimatize for 1 week. During this time, a set of four spare mesocosms containing 4 mg N‐total and the same bacterial community were used to collect mosquito larvae to be used in the experimental set‐up.

Evaporated water was replenished daily using dechlorinated tap water stored at ambient temperature. To maximally mimic field conditions and to limit the amount of stress of the predators, natural shelter for newts in the form of a handful of Canadian waterweeds (*Elodea vulgaris*), a stone to climb out of the water, and an air stone connected to an air pump (Vt AP‐10) was provided in each mesocosm. Prey densities of 50 third/fourth instar mosquito larvae were added to each mesocosm.

Mosquito larvae were counted by carefully and partially removing the mesh lid as to not disturb any predators or mosquito larvae that had emerged. Any adult mosquitoes were captured using an aspirator and excluded from further counts. The lid was then fully removed, and mosquito larvae were subsequently counted by visual inspection of the top of the water column.

#### 2.1.2 Experiment 1: Relative Predator Effectiveness

Kill rates of different amphibian versus aquatic insect predators were assessed in May 2020. The experiment consisted of four predator treatments, with either one individual of *L. vulgaris* , *A. bipustulatus* , *N. glauca* , or no predator (control). Each treatment had three replicates. The experiment took place during May 2020. The number of mosquito larvae, pupae, and adults were counted 1, 2, and 3 h after the (50) mosquito larvae were placed in the mesocosm.

#### 2.1.3 Experiment 2: Predation Across Eutrophication

The impact of eutrophic conditions on predator effectiveness was assessed by taking two eutrophication treatments (4 mg/L N‐total and 8 mg/L N‐total) and two predation treatments ( *L. vulgaris* , control), with eight *L. vulgaris* replicates and four non‐predator control replicates. The experiment took place at the beginning of May 2020 and had a duration of 3 days. The number of mosquito larvae, pupae, and adults was counted 10 times at the following time points: 1, 2, 4, 6, 12, 14, 16, 18, 36, 38, 40, 42, 52, and 62 h after *L. vulgaris* had been placed in the mesocosm with the (50) mosquito larvae. We performed daily chlorophyll a and turbidity measurements using an Aquafluor 8000–010 using manufacturers' protocols, as these are indicators for both visibility and resource competition due to their relation with bacterial and algal metabolism (Ansa‐Asare et al. 2000; Coolidge 2017).

#### 2.1.4 Experiment 3: Sex‐Specific Amphibian Predator Effectiveness

**FIGURE 1:**
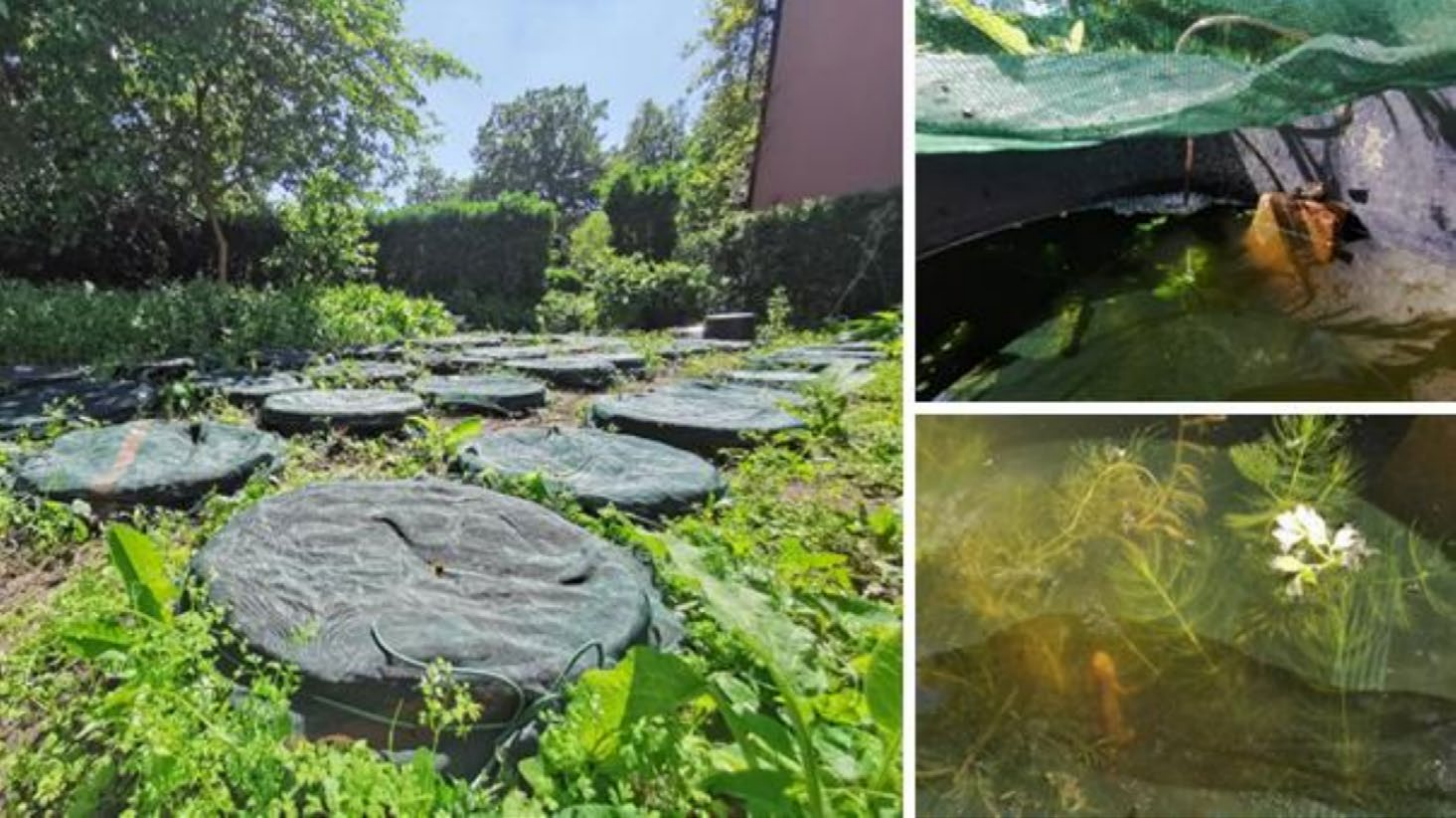
Overview of experimental setup for amphibian predator effectiveness; experiment 3 (left). Contents of the mesocosms are shown on the right including the stone as a resting spot and air stone for oxygen (top right), and waterweeds as natural shelter (bottom right).

Amphibian predator effectiveness was assessed by imposing three predation treatments ( *L. vulgaris* , *P. esculentus* , control). Due to limitations in availability, we used two male and nine female replicates for *L. vulgaris* , seven male and four female replicates for *P. esculentus* , and fourteen control replicates. The experiment took place in June 2021. A terrestrial resting spot was included in each mesocosm in the form of a 180 × 87 × 41 mm brick placed vertically in the water (Figure 1). To detect potential differences in temperature as a result of (partial) tree‐shade, temperature was measured using an iButton near the water surface at the corners and middle row perpendicular to a neighboring tree (mesocosms 1, 5, 9, 14, 23, 28, 32, and 36). The number of mosquito larvae, pupae, and adults were counted 10 times at the following time points: 1, 2, 4, 8, 24, 28, 32, 48, 56, and 72 h. The experiment started when the mosquito larvae were added, 40 h after the predators had been collected.

The experiment was repeated directly after the first round to determine whether the 40‐h food deprivation had affected predatory behavior. This second round was performed in triplicate, for which a selection of the predators was used, with two male and one female replicate for *P. esculentus* and three female replicates for *L. vulgaris* . These individuals were taken from those used during the previous round. Other predators were removed from the setup and placed in the setup for the comparisons to other genera.

#### 2.1.5 Experiment 4: Oviposition Behavior

Non‐consumptive impacts of predator cues on mosquito oviposition behavior were assessed in the same experimental setup as in experiment 3. After the second round of the main experiment, the mesh lids of all experimental mesocosms were taken off and all remaining predators were removed. As such, the experiment consisted of two male and nine female replicates for *L. vulgaris* , seven male and four female replicates for *P. esculentus* , and fourteen control replicates. The water from each mesocosm was strained through a 300 μm sieve to remove any remaining larvae. The mesocosms were then left open for 2 weeks during which egg rafts were counted and removed daily. The water in each mesocosm was filtered daily using a 300 μm sieve to prevent colonization by other (predator) species.

#### 2.1.6 Author's Personal Observation: Comparison With Other Mosquito Genera

Predatory behavior on the mosquito genera *Anopheles* and *Aedes* was assessed using a glass 40 × 30 × 30 cm aquarium filled with 20 L low eutrophic (4 mg/L N total) water. Two *L. vulgaris* or two *P. esculentus* were placed in the aquarium and left to acclimate for 5 min. Three larvae of either *Aedes* sp. or *Anopheles maculipennis* were then added, and feeding was recorded for 5 min, after which the remaining mosquito larvae were removed using plastic pipettes. The predators were then collected and released at their capture location. This was repeated until all larvae had been eaten. Due to limited availability, only seven *Aedes* larvae and five *Anopheles* larvae were used in total.

### 2.2 Rearing of Mosquito Larvae

Egg rafts of *Cx. pipiens* were collected during 2 weeks prior to the start of an experiment at the experimental site. To this end, three 8 L black polypropylene buckets were filled with three liters hypertrophic water, which has been shown to be an attractive oviposition environment to female *Cx. pipiens* (100 mg N‐total/L; Boerlijst et al. 2023), after which they were placed under tree cover. The larvae were subsequently allowed to hatch in the buckets, where they were kept at ambient temperature until the start of the experiment. Previous studies indicated that the used conditions attract *Cx. pipiens* s.l. and *Culiseta annulata* only (Boerlijst et al. 2023; Dellar et al. 2022). The collected egg rafts were distinguished from those of *Culiseta annulata* by their difference in size (Chapman et al. 2020; Sames et al. 2005).

### 2.3 Sourcing Predators

All predators were captured from the neighboring lake of the Living Lab field station using an aquatic net. The two aquatic insect species, *A. bipustulatus* and *N. glauca* , were collected on the day of the experiment and were identified using the Freshwater Life field guide (Greenhalgh and Ovenden 2007) whilst making sure all individuals were of similar size. The two amphibian species, *L. vulgaris* and *P. esculentus*, were collected during the 2 days prior to the experiment. All predators were kept individually in the experimental setup until the start of the experiment to prevent cannibalistic behavior.

### 2.4 Statistical Analysis

All data were analyzed in R version 4.3.2 (R Core Team 2018). Linear (mixed effects) models were used to test for differences in predator effectiveness across the experiments. Box‐cox transformation was applied when it improved normality and variance homogeneity of the residuals (Table 2). All models (Table 2), including random effects, were optimized by Akaike information criterion using backwards selection from a full model with interactions between all terms. This full model included covariates like temperature, chlorophyll‐a and turbidity. Dependent variables were tested for normality and assessed using Quantile Quantile‐plots and a Levene's test (*p* = 0.05). Power and effect size were calculated using the anova_stats function from the sjstats package version 0.19.0 (Table S1).

**TABLE 2:** Linear model after selection per experiment.

Experiment	Linear model	Lambda
1. Relative predator effectiveness	Mortality ~ Predator	0.626
2. Predation across eutrophication	Mortality ~ Eutrophication + Hours	
3. Sex‐specific amphibian predator effectiveness	Mortality ~ Predator × Hours + Temperature + Error (Mesocosm/(Predator × Hours))	0.62 (experiment 3.1) 0.59 (experiment 3.1)
4. Oviposition behavior	Egg rafts ~ Predator × Day + Error (Mesocosm/Day)	NA

*Note:* “Cosm” refers to the container or experimental unit. Predator refers to the species used.

Absolute mortality rates were used for statistical analysis, whereas the proportion of mortality due to predation was used for visualization purposes. The proportions of mortality due to predation were calculated by subtracting the background mortality, here defined as the mean mortality in the control group at that moment. As such, variance in control is not visualized, but was evaluated and thus accounted for.

## 3 Results

### 3.1 Experiment 1: Assessing Relative Predator Effectiveness

**FIGURE 2:**
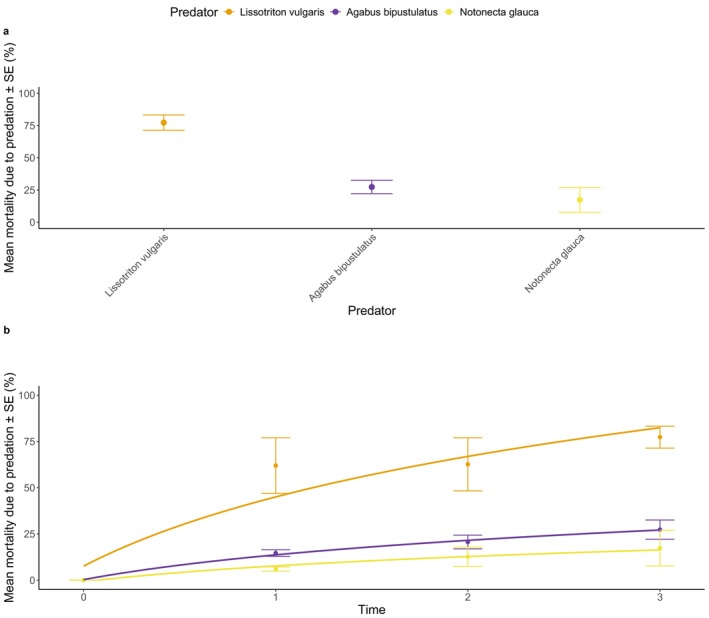
Proportion of mortality due to predation (%) per predator species (Experiment 1) at the end of the experiment (a), and over time including a log‐fit trend‐line (b).

All predators successfully captured and consumed mosquito larvae (Figure 2; Table S1). No difference in predator effectiveness was detected between *A. bipustulatus* and *N. glauca* at the end of the experiment (*t*(3,8) = −0.985, *p* > 0.05, partial η^2^ = 0.173, power = 1). However, differences between the amphibian and aquatic insect predators were found as *L. vulgaris* consumed on average 2.8 (and up to four) times as many larvae as *A. bipustulatus* (t(3,8) = −4.924, *p* < 0.01, partial η^2^ = 0.909, power = 1) and on average 4.5 (and up to eight) times as many larvae as *N. glauca* (t(3,8) = −5.909, *p* < 0.001, partial η^2^ = 0.876, power = 1).

#### 3.1.1 Experiment 2: Predation Across Eutrophication

*Lissotriton vulgaris* was successful in capturing mosquito larvae irrespective of eutrophication level (t(3,92) = −1.814, *p* > 0.05, partial η^2^ = 0.008, power = 0.136; Table S2), and their kill rate increased over time (t(9,92) = 13.545, *p* < 0.001, partial η^2^ = 0.666, power = 1).

### 3.2 Experiment 3: Sex‐Specific Amphibian Predator Effectiveness

#### 3.2.1 Round 1: Short‐Term Food Deprivation

**FIGURE 3:**
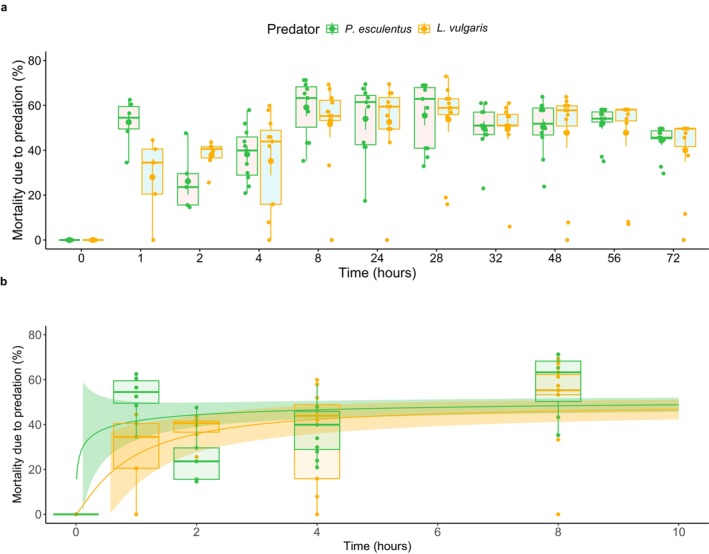
Proportion of mortality due to predation (%) over time (after 40‐h food deprivation; Experiment 3.1) per predator type depicted as boxplot with outliers as dots (a) and dose–response curve with standard error up until the asymptote is reached (b). Both sexes are represented as no differences were detected. Both absolute mortality and the proportion of mortality due to predation were calculated using counts per mesocosm per sampling moment. As these manual observations may be imperfect, visualization may show “dips” in mortality. This is accounted for in the statistical analysis by using a fitted model.

After a 40‐h food deprivation, approximately half of the (50) mosquito larvae within the experiment were eaten after 1–2 h, which steadily progressed during subsequent hours (f(10,266) = 200.948, *p* < 0.001, partial η^2^ = 0.875, power = 1; Figure 3; Table S3) and differed between predator treatment (f(20,266) = 5.253, *p* < 0.001, partial η^2^ = 0.282, power = 1). A minor increase of predation rates with temperature (range 15.5°C–21.5°C) was detected (f(1,266) = 8.279, *p* < 0.01, partial η^2^ = 0.031, power = 0.835). Post hoc analysis showed higher mosquito mortality for *P. esculentus* (*t* = −4.016, *p* < 0.01) and *L. vulgaris* (*t* = −2.667, *p* < 0.05) compared to the control from 2 h onwards. No difference between the two predator species was detected (*t* = 1.195, *p* > 0.05). No difference between sexes was detected (Figure S1).

#### 3.2.2 Round 2: No Short‐Term Food Deprivation

**FIGURE 4:**
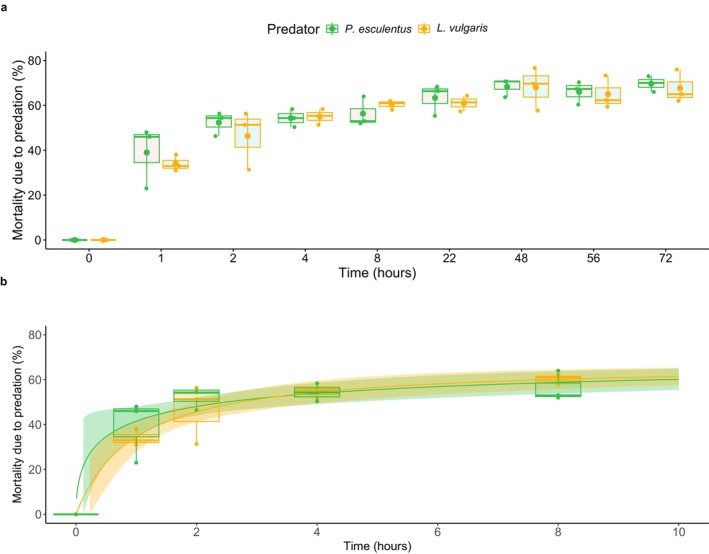
Proportion of mortality due to predation (%) over time per predator type without food deprivation (Experiment 3.2), depicted as boxplot with outliers as dots (a) and dose–response curve with standard error up until the asymptote is reached (b). Both sexes are represented as no differences were detected. Both absolute mortality and proportion of mortality due to predation were calculated using counts per mesocosm at each moment. As these manual observations may be imperfect, visualization may show “dips” in mortality. This is accounted for in the statistical analysis by using a fitted model.

Similar kill rates were found without 40‐h food deprivation, with the majority of the prey consumed during the first 2 h. Mosquito mortality increased over time (f(8,35) = 147.777, *p* < 0.001, partial η^2^ = 0.965, power = 1; Figure 4; Table S4) per predator treatment (f(16,35) = 8.977, *p* < 0.001, partial η^2^ = 0.763, power = 1). No effect of temperature was detected (f(13,35) = 0.647, *p* > 0.05, partial η^2^ = 0.744, power = 1). Post hoc analysis showed higher mosquito mortality for *P. esculentus* (t = −15.251, *p* < 0.001) and *L. vulgaris* (*t* = −15.163, *p* < 0.001) compared to the control from 1 h onwards. No difference between the two predator species was detected (*t* = 0.406, *p* > 0.05). No difference between sexes was detected (Figure S2).

### 3.3 Experiment 4: Oviposition Behavior

During the 2 week period, a total of 32 mosquito egg‐rafts were laid, of which 27 in the control treatment, and the remaining 5 in mesocosms that had been occupied by newts (Figure S3; Table S5). All rafts were deposited in the mesocosms located at the border of the experimental setup. Differences were detected across the predator treatments (*Χ* ^2^ = 24.86, df = 6, *p* < 0.001, Kramers‐v = 0.19, power = 0.82). Post hoc analysis revealed differences between the control and *L. vulgaris* (*Χ* ^2^ = 8.63, df = 3, *p* < 0.05) and between *L. vulgaris* and *P. esculentus* (*Χ* ^2^ = 18.95, df = 3, *p* < 0.001).

### 3.4 Author's Personal Observation: Comparisons to Other Genera

The experiment to confirm whether other representative species from other mosquito genera were also consumed by the same predators was successful and confirmed that all 7 *Aedes* and 5 *Anopheles* larvae were eaten (Video S1).

## 4 Discussion

The aim of this study was to assess the potential of amphibians to control mosquitoes, as part of a One Health approach, by determining the predator effectiveness of a range of relevant mosquito predators in temperate regions. Here, we report effects on different mosquito life stages, both during larval development and oviposition. Both amphibians ( *L. vulgaris* and *P. esculentus* ) exhibited similar and notably high larval consumption of *Cx. pipiens*, regardless of preceding food deprivation. *L. vulgaris* consumed on average 2.8 (and up to four) times as many larvae as *A. bipustulatus* and on average 4.5 (and up to eight) times as many larvae as *N. glauca* . Additionally, breeding sites with prior amphibian presence deterred egg‐laying, also for neighboring breeding sites. These deterrent effects appear more significant and ecologically diverse than previously reported (Mokany and Shine 2003; Rubbo et al. 2011), adding to the evidence that ephemeral wetlands with an abundance of natural predators might effectively reduce mosquito populations without the need for ecologically harmful larvicides (Dale and Knight 2012).

To date, most interventions to suppress mosquito populations involve the use of chemicals like organophosphates, neonicotinoids and pyrethroids. However, these chemical methods are short‐term solutions, leading to resistance (Hamdan et al. 2005; Li et al. 2002) and creating a pesticide treadmill undermining ecosystem health. As competing species are often impacted similarly, chemical control may result in rapid recolonization (Meyabeme Elono et al. 2018) and overcompensation (Juliano 2007; Neale and Juliano 2019) by mosquitoes and ecological imbalances (Allgeier et al. 2019; Brühl et al. 2020; Meyabeme Elono et al. 2018), posing risks, especially to natural ecosystems like conservation areas. This combined with the limited information on food‐web effects (Brühl et al. 2020) and the subsequent unknown fate of the substances makes its use in conservation areas risky. In contrast, biological control, using native, naturally occurring predators, presents a less problematic approach. By actively releasing or facilitating native predators, it may offer a more sustainable and preventive strategy. This method deters mosquito egg‐laying (Rubbo et al. 2011; Sougué et al. 2021) and limits immature mosquito survival whilst lowering their growth rates, fecundity and delaying reproduction (Fischer et al. 2012; Lundkvist et al. 2003; Schrama et al. 2018). Additionally, stressful conditions—including predator presence—promote a more heterogeneous mix of developmental stages, as a result of different growth strategies among the mosquito larvae (Fischer et al. 2012; Knight et al. 2004), which could enable cannibalistic behavior (El Husseiny et al. 2018; Koenraadt and Takken 2003). Indeed, results from our study highlight the importance of seriously considering such measures, not only because they are likely cost‐effective, but also because they may be far more sustainable than chemical alternatives.

An important remaining question is how these effects translate into more ecologically complex settings. Namely, while biological control presents numerous advantages, its potential as a truly effective control method in natural mosquito breeding sites still requires further investigation. Natural ephemeral ecosystems may be more biologically and physiochemically complex than the mesocosm setup used. Although the current results show clear trends, this added complexity could lower the magnitude of the real‐world impacts. For instance, open, natural water bodies experience continuous potential recolonization, possibly leading to more spatially heterogeneous outcomes as compared to the current mesocosms, depending on specifics like vegetation structure. Similarly, the deterrent impacts on ovipositing behavior may be more transient when predator cues can be displaced or mixed over a larger area. Additionally, the current experiments did not consider alternative prey, such as chironomids, which are commonly found alongside mosquito larvae (Dinithi and Hemantha 2020; Leisnham et al. 2007; Talaga et al. 2020). Therefore, this study does not account for the effects of prey preference. However, both currently assessed amphibian species hunt opportunistically (Kovács et al. 2014; Roşca et al. 2013). As mosquito larvae spend most of their time at the top of the water column (Becker et al. 2010), consistently dive as anti‐predator behavior (Awasthi et al. 2012), and often occur at high densities, they are considered easy prey. It could be suggested that they would provide a prominent food source if available, even in complex communities, which is confirmed by their relative abundance in the stomach contents of both amphibian species (Brodman and Dorton 2006; Tyler 1958). Moreover, *Cx. pipiens* often selects for breeding habitats with minimal competition and predation (Alcalay et al. 2019; Dhileepan 1997), which further reduces potential effects of prey preference on predator behavior.

Similarly, the current study evaluated predator effectiveness at the individual level, without considering the effect of relative predator densities. Aquatic insect predators may be present in much higher densities than amphibians. Consequently, although *L. vulgaris* and *P. esculentus* individuals consume more larvae, this effect may be less pronounced at the community level. Especially when re‐evaluating predator effectiveness across average predator biomass, that is, larvae consumed per gram (Table S6), aquatic insect predators seem much more effective in the short term. However, amphibians, which can readily move between water bodies and have stomachs capable of digesting large amounts of prey simultaneously (Bissattini et al. 2021; Brodman and Dorton 2006; Tyler 1958), may still prove to be important predators in influencing mosquito populations over longer periods.

Our results suggest that tested amphibians are highly effective mosquito predators, irrespective of species, without discernible differences in kill rates between sexes, among similarly sized individuals of amphibians, or among aquatic insect predators. When applying these findings to other mosquito groups, it is essential to consider that mosquito larvae exhibit species‐specific feeding behaviors that influence their position in the water column (Dadd 1975; Merritt et al. 1992). Understanding these behaviors is crucial for assessing predator effectiveness, particularly in scenarios of static predatory behavior, necessitating comprehensive testing across diverse mosquito groups and predator species. However, it is noteworthy that a predominant anti‐predator response among most mosquito larvae is diving (Awasthi et al. 2012; Sih 1986). Notably, we found that *L. vulgaris* effectively preys on larvae of *Culex*, *Aedes*, and *Anopheles*, primarily in proximity to the container bottom, to which the larvae flee upon disturbance. *Pelophylax esculentus* was found to sit and wait floating at the water surface, we hypothesize until the larvae re‐emerge after diving, consistent with previous literature (Anamaria et al. 2011; Kovács et al. 2014). As such, it is probable that our findings also translate to similar predation rates for other mosquito species in similar ephemeral water bodies.

When considering the effectiveness of predation under different abiotic conditions, it is important to recognize their potential interactions (Krol et al. 2019). Typically, murky and nutrient‐rich waters have a positive impact on larval mosquito feeding behavior (Dadd 1975; Merritt et al. 1992), thus benefitting *Cx. pipiens'* survival (Boerlijst et al. 2023). Hence, it was expected that eutrophication would affect the capacity of predators to kill mosquito larvae (Schmutzer et al. 2008), which could operate either through lowered oxygen acquisition (Coffin et al. 2018), lowered hunting efficiency due to increased anti‐predator behavior (Tuno et al. 2004), or reduced vision‐based hunting (Abrahams and Kattenfeld 1997). Surprisingly, eutrophication levels did not affect predation levels, with *L. vulgaris* successfully capturing mosquito larvae regardless, suggesting unaffected predation behavior, possibly mediated due to alternative olfactory or tactile cues (Ranta et al. 1990).

The observed effects of predators on avoidance of egg‐laying by mosquitoes may ultimately be even more important than the direct effects of larval killing, suggesting that mosquito predators are successful in establishing a mosquito landscape‐of‐fear (Brown et al. 1999). Ovipositing rates were relatively low, as adult female mosquitoes mostly refused to deposit eggs anywhere near our experimental setup. This effect contrasted with the days before experiment 3.1, when the experimental setup was used to collect the mosquito larvae to be used as prey. Similarly, the impacts were far stronger than the deterrence by abiotic factors such as salt, nutrient availability, or temperature under similar (abiotic) conditions (Boerlijst et al. 2023, 2024), or relative to some aquatic insect predators (Eitam and Blaustein 2004; Vonesh and Blaustein 2010; Why et al. 2016). This may be a result of increased selection pressure for predator avoidance among *Culex* due to their “all‐or‐nothing” raft‐laying strategy (Day 2016; Vonesh and Blaustein 2010), which insinuates that further exploration is needed before these results may be extrapolated to species that use other egg‐laying strategies like *Aedes* and *Anopheles*. Nevertheless, for *Culex*, these results indicate that these predators remain in control, even in between meals and during temporary absence.

Overall, our results suggest that amphibian predators may have important long‐term negative effects both on mosquito larval and egg stages. However, relative abundances of predator species play a crucial role in their effectiveness, meaning that despite the amphibians' higher effectiveness, their overall impact could be mitigated by their lower densities when compared to aquatic insects. Similarly, the increased complexity of open, natural systems may lower the magnitude of the real‐world impacts. Still, given their substantial impact on oviposition rates and their ability to readily move over land to colonize temporary mosquito breeding habitats, amphibians are likely relevant actors in controlling mosquito larval populations for relatively small (peri‐)urban habitats. As such, facilitating endemic amphibians and endemic mosquito predators at large in wetlands and anthropogenic landscapes like urban blue may prove to be a valuable and effective component of One Health approaches to mosquito control.

## Author Contributions

**S. P. Boerlijst:** conceptualization (equal), data curation (equal), formal analysis (lead), investigation (lead), methodology (equal), project administration (lead), visualization (lead), writing – original draft (lead), writing – review and editing (lead). **A. Ummels:** conceptualization (equal), formal analysis (supporting), investigation (equal), methodology (equal), writing – review and editing (equal). **A. M. Spitzen‐van der Sluijs:** conceptualization (equal), formal analysis (supporting), writing – review and editing (equal). **J. Spitzen:** conceptualization (equal), investigation (supporting), writing – review and editing (equal). **R. W. Bouman:** formal analysis (equal), writing – review and editing (equal). **E. Boelee:** funding acquisition (equal), investigation (supporting), supervision (equal), writing – review and editing (equal). **P. M. van Bodegom:** formal analysis (equal), funding acquisition (equal), investigation (equal), supervision (equal), writing – review and editing (equal). **M. Schrama:** conceptualization (equal), formal analysis (equal), funding acquisition (equal), supervision (equal), writing – original draft (equal), writing – review and editing (equal).

## Funding

This publication is part of the project “Preparing for vector‐borne virus outbreaks in a changing world: a One Health Approach” (NWA.1160.1S.210) which is (partly) financed by the Dutch Research Council (NWO).

## Ethics Statement

All experiments were performed under supervision of the RAVON foundation. The Defense Safety Inspectorate deemed the experiments to be exempt of the animal experiment legislation as the discomfort inflicted was negligible since (i) the displacement was within 6 m for less than 10 days, (ii) there was no (medical) intervention, (iii) there was no long‐term food deprivation, and (iv) they were released into their original habitat.

## Conflicts of Interest

The authors declare no conflicts of interest.

## Supporting information


**Video S1:** Feeding behavior of smooth newt ( *Lissotriton vulgaris* ) in response to (anti‐predator response of) Aedes and Anopheles mosquito larvae.

**Appendix S1:** Supporting Information.

## Data Availability

The original datasets used and analyzed during the present study are freely and openly available within the Zenodo repository https://doi.org/10.5281/zenodo.11128264 under reference number 10.5281/zenodo.11128264.
