# Supplementary material for: Clashing in Murky Waters: On Amphibian Mosquito Suppression
Source: Ecol Evol. 2026 Mar 7;16(3):e73127. doi: 10.1002/ece3.73127 (PMC12966807; doi:10.1002/ece3.73127)
Supplement: Supplementary file 2 — Appendix S1: Supporting Information. [file ECE3-16-e73127-s001.docx]

**Electronic** **appendix**


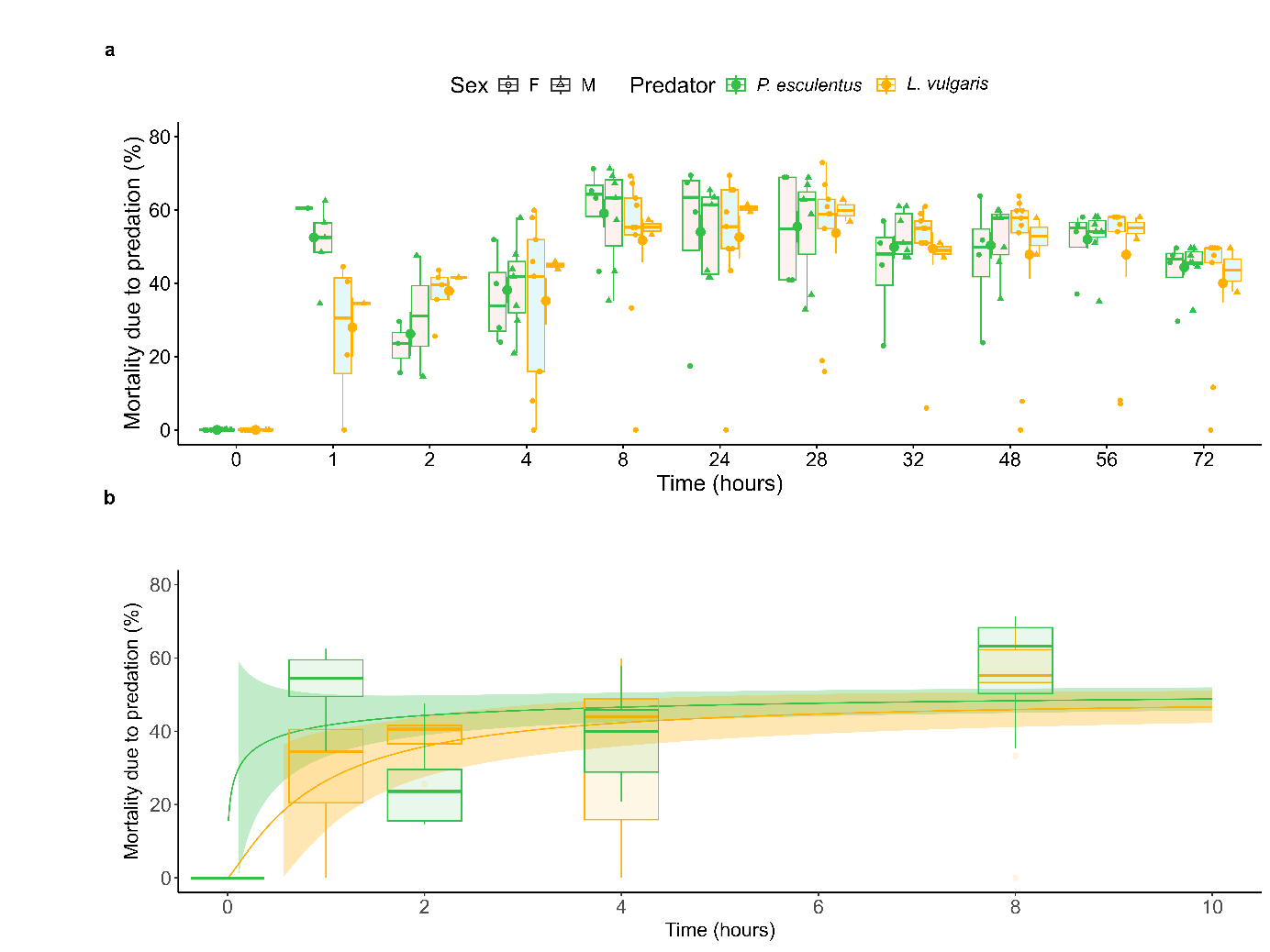


**Figure** **S1** Proportion of mortality due to predation (%) over time (after 40h fast) per predator type depicted as (a) boxplot per sex with outliers as dots and (b) dose-response curve with standard error up until the asymptote is reached. Predator sex is indicated by a circle for female, triangle for male.


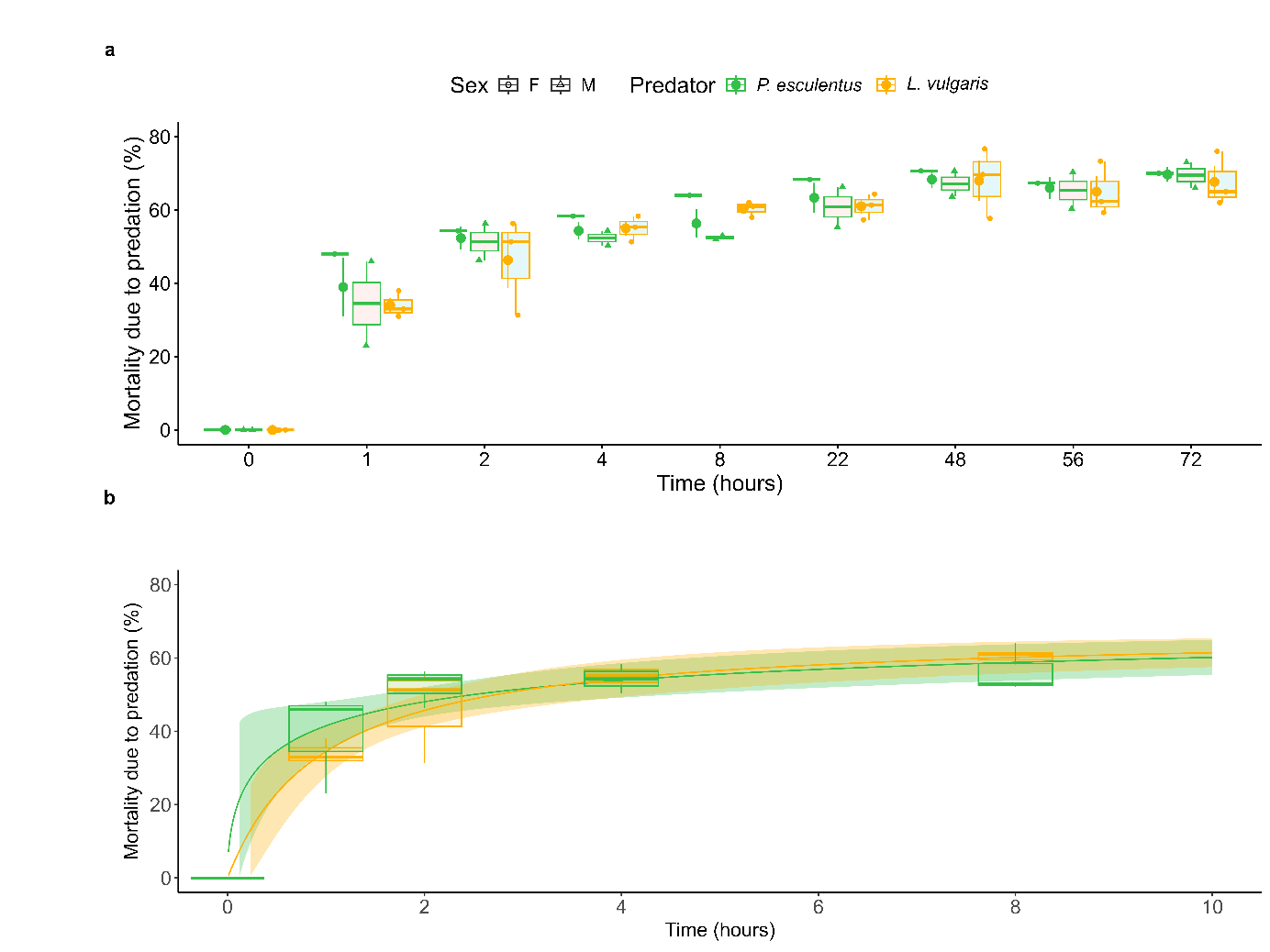


**Figure** **S2** Proportion of mortality due to predation (%) over time per predator type without fast depicted as (a) boxplot per sex with outliers as dots and (b) dose-response curve with standard error up until the asymptote is reached. Predator sex is indicated by a circle for female, triangle for male.

**
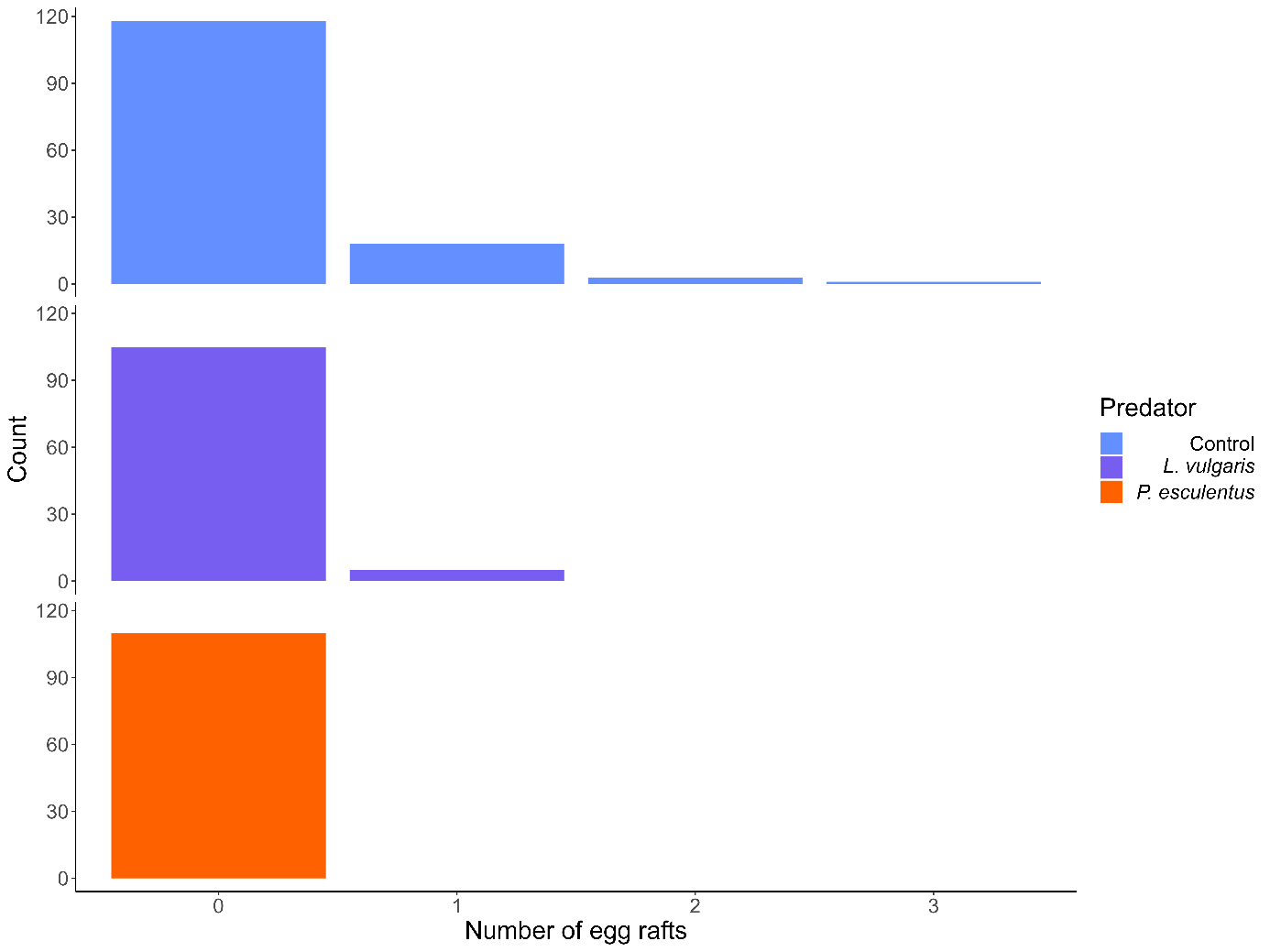
**

**Figure** **S3** Oviposition behavior depicted as the number of egg-rafts counted per day per predator treatment over the two weeks.

**Table S1a** Anova table experiment 1

|  | Estimate | Std. Error | t value | Pr(>\|t\|) | Signif |
| --- | --- | --- | --- | --- | --- |
| (Intercept) | 77.33 | 7.18 | 10.770 | 3.79e-05 | *** |
| PredatorAgabus bipustulatus | -50.00 | 10.15 | -4.924 | 0.00265 | ** |
| PredatorNotonecta glauca | -60.00 | 10.15 | -5.909 | 0.00105 | ** |

Signif. codes: 0 ‘***’ 0.001 ‘**’ 0.01 ‘*’ 0.05 ‘.’ 0.1 ‘ ’ 1

**Table S1b** Power analysis experiment 1

Total dataset

| term | df | Sum sq | Mean sq | statistic | p.value | Eta sq | Partial etasq | Omega sq | Partial omega sq | Epsilon sq | Cohens f | power |
| --- | --- | --- | --- | --- | --- | --- | --- | --- | --- | --- | --- | --- |
| Predator | 2 | 6200 | 3100.000 | 20.043 | 0.002 | 0.870 | 0.870 | 0.809 | 0.809 | 0.826 | 2.585 | 1 |
| Residuals | 6 | 928 | 154.667 |  |  |  |  |  |  |  |  |  |

*L. vulgaris* vs *N. glauca*

| term | df | Sum sq | Mean sq | statistic | p.value | Eta sq | Partial eta sq | omegasq | Partial omega sq | Epsilon sq | Cohens f | power |
| --- | --- | --- | --- | --- | --- | --- | --- | --- | --- | --- | --- | --- |
| Predator | 1 | 5400.000 | 5400.000 | 28.223 | 0.006 | 0.876 | 0.876 | 0.819 | 0.819 | 0.845 | 2.656 | 0.997 |
| Residuals | 4 | 765.333 | 191.333 |  |  |  |  |  |  |  |  |  |

*L. vulgaris* vs *A. bipustulatus*

| term | df | Sum sq | Mean sq | statistic | p.value | Eta sq | Partial eta sq | Omega sq | Partial omega sq | Epsilon sq | Cohens f | power |
| --- | --- | --- | --- | --- | --- | --- | --- | --- | --- | --- | --- | --- |
| Predator | 1 | 3750.000 | 3750.000 | 40.179 | 0.003 | 0.909 | 0.909 | 0.867 | 0.867 | 0.887 | 3.169 | 1 |
| Residuals | 4 | 373.333 | 93.333 |  |  |  |  |  |  |  |  |  |

*N. glauca* vs. *A. bipustulatus*

| term | df | Sum sq | Mean sq | statistic | p.value | Eta sq | Partial eta sq | Omega sq | Partial omega sq | Epsilon sq | Cohens f | power |
| --- | --- | --- | --- | --- | --- | --- | --- | --- | --- | --- | --- | --- |
| Predator | 1 | 150.000 | 150.000 | 0.836 | 0.412 | 0.173 | 0.173 | -0.028 | -0.028 | -0.034 | 0.457 | 0.141 |
| Residuals | 4 | 717.333 | 179.333 |  |  |  |  |  |  |  |  |  |

**Table S2a** Anova table experiment 2

|  | Estimate | Std. Error | t value | Pr(>\|t\|) | Signif |
| --- | --- | --- | --- | --- | --- |
| (Intercept) | -4.27542 | 13.15893 | -0.325 | 0.7488 |  |
| Control + extra eutrophic | -1.62077 | 2.07676 | -0.780 | 0.4448 |  |
| Smooth newt + eutrophic | -26.25201 | 1.72704 | -15.201 | 4.36e-12 | *** |
| Smooth newt + extra eutrophic | -28.55619 | 1.73145 | -16.493 | 1.03e-12 | *** |
| Eutrophication | 0.11022 | 0.04109 | 2.682 | 0.0147 | * |

Signif. codes: 0 ‘***’ 0.001 ‘**’ 0.01 ‘*’ 0.05 ‘.’ 0.1 ‘ ’ 1

**Table S2b** Power analysis experiment 2

| term | df | Sum sq | Mean sq | statistic | P value | Eta sq | Partial eta sq | Omega sq | Partial omega sq | Epsilon sq | Cohens f | power |
| --- | --- | --- | --- | --- | --- | --- | --- | --- | --- | --- | --- | --- |
| Eutrophication | 1 | 282.095 | 282.095 | 0.723 | 0.397 | 0.003 | 0.008 | -0.001 | -0.003 | -0.001 | 0.088 | 0.136 |
| Time | 1 | 71588.013 | 71588.013 | 183.469 | <0.001 | 0.662 | 0.664 | 0.656 | 0.655 | 0.658 | 1.405 | 1.000 |
| Residuals | 93 | 36287.850 | 390.192 |  |  |  |  |  |  |  |  |  |

**Table S3** Anova table experiment 3 round 1

|  | Df | Sum Sq | Mean Sq | F value | Pr(>F) | Signif |
| --- | --- | --- | --- | --- | --- | --- |
| Time | 10 | 47.07 | 4.707 | 200.948 | < 2e-16 | *** |
| Temperature | 1 | 0.19 | 0.194 | 8.279 | 0.00434 | ** |
| Predator:Time | 20 | 2.46 | 0.123 | 5.253 | 4.15e-11 | *** |
| Residuals | 266 | 6.23 | 0.023 |  |  |  |

Signif. codes: 0 ‘***’ 0.001 ‘**’ 0.01 ‘*’ 0.05 ‘.’ 0.1 ‘ ’ 1

**Table S3b** Power analysis experiment 3 round 1

| stratum | term | df | Sum sq | Mean sq | statistic | P value | Eta sq | Partial eta sq | Omega sq | Partial omega sq | Epsilon sq | Cohens f | power |
| --- | --- | --- | --- | --- | --- | --- | --- | --- | --- | --- | --- | --- | --- |
| Cosm | Predator | 2 | 16.567 | 8.283 | 17.089 | <.001 | 0.189 | 0.727 | 0.188 | 0.681 | 0.188 | 1.631 | 1.000 |
| Cosm | Time | 1 | 0.605 | 0.605 | 1.248 | 0.274 | 0.007 | 0.088 | 0.007 | 0.070 | 0.007 | 0.312 | 0.999 |
| Cosm | Temperature | 1 | 1.199 | 1.199 | 2.473 | 0.128 | 0.014 | 0.161 | 0.013 | 0.132 | 0.013 | 0.439 | 1.000 |
| Cosm | Predator:Time | 2 | 0.739 | 0.369 | 0.762 | 0.477 | 0.008 | 0.106 | 0.008 | 0.082 | 0.008 | 0.344 | 1.000 |
| Cosm | Residuals | 26 | 12.603 | 0.485 |  |  | 0.144 | 0.669 | 0.137 | 0.608 | 0.137 | 1.422 | 1.000 |
| Cosm:Time | Time | 10 | 47.070 | 4.707 | 200.948 | <.001 | 0.537 | 0.883 | 0.534 | 0.858 | 0.534 | 2.749 | 1.000 |
| Cosm:Time | Temperature | 1 | 0.194 | 0.194 | 8.279 | 0.004 | 0.002 | 0.030 | 0.002 | 0.022 | 0.002 | 0.176 | 0.821 |
| Cosm:Time | Predator:Time | 20 | 2.461 | 0.123 | 5.253 | <.001 | 0.028 | 0.283 | 0.023 | 0.205 | 0.023 | 0.628 | 1.000 |
| Cosm:Time | Residuals | 266 | 6.231 | 0.023 |  |  |  |  |  |  |  |  |  |

Table S3c Mean background mortality rates experiment 3 round 1. This includes larvae that emerged during the time of the experiment.

| **Time** | **Absolute mortality rate control** |
| --- | --- |
| 0 | 0 |
| 1 | 0.195 |
| 2 | 0.324 |
| 4 | 0.2809091 |
| 8 | 0.2472727 |
| 24 | 0.3054545 |
| 28 | 0.2709091 |
| 32 | 0.37 |
| 48 | 0.3618182 |
| 56 | 0.4190909 |
| 72 | 0.5036364 |

**Table S4** Anova table experiment 3 round 2

|  | Df | Sum Sq | Mean Sq | F value | Pr(>F) | Signif |
| --- | --- | --- | --- | --- | --- | --- |
| Time | 8 | 10.702 | 1.3377 | 147.777 | < 2e-16 | *** |
| Temperature | 1 | 0.006 | 0.0059 | 0.647 | 0.425 |  |
| Predator:Time | 16 | 1.300 | 0.0813 | 8.977 | 1.8e-09 | *** |
| Residuals | 47 | 0.425 | 0.0091 |  |  |  |

Signif. codes: 0 ‘***’ 0.001 ‘**’ 0.01 ‘*’ 0.05 ‘.’ 0.1 ‘ ’ 1

**Table S4b** Power analysis experiment 3 round 2

| stratum | term | df | Sum sq | Mean sq | statistic | P value | Eta sq | Partial eta sq | Omega sq | Partial omega sq | Epsilon sq | Cohens f | power |
| --- | --- | --- | --- | --- | --- | --- | --- | --- | --- | --- | --- | --- | --- |
| Cosm | Predator | 2 | 4.773 | 2.387 | 160.229 | <.001 | 0.503 | 0.962 | 0.502 | 0.937 | 0.502 | 5.057 | 1.000 |
| Cosm | Temperature | 1 | 0.000 | 0.000 | 0.004 | 0.954 | 0.000 | 0.000 | 0.000 | -0.012 | 0.000 | 0.017 | 0.052 |
| Cosm | Residuals | 5 | 0.074 | 0.015 |  |  | 0.008 | 0.285 | 0.006 | 0.145 | 0.006 | 0.632 | 0.938 |
| Cosm: Time | Time | 8 | 3.676 | 0.460 | 115.728 | <.001 | 0.388 | 0.952 | 0.384 | 0.919 | 0.384 | 4.438 | 1.000 |
| Cosm: Time | Temperature | 1 | 0.009 | 0.009 | 2.344 | 0.132 | 0.001 | 0.048 | 0.001 | 0.016 | 0.001 | 0.223 | 0.334 |
| Cosm: Time | Predator: Time | 16 | 0.767 | 0.048 | 12.067 | <.001 | 0.081 | 0.804 | 0.074 | 0.686 | 0.074 | 2.027 | 1.000 |
| Cosm: Time | Residuals | 47 | 0.187 | 0.004 |  |  |  |  |  |  |  |  |  |

Table S4c Mean background mortality rates experiment 3 round 2. This includes larvae that emerged during the time of the experiment.

| **Time** | **Absolute mortality rate control** |
| --- | --- |
| 0 | 0 |
| 1 | 0.12 |
| 2 | 0.086667 |
| 4 | 0.096667 |
| 8 | 0.14 |
| 22 | 0.206667 |
| 48 | 0.233333 |
| 56 | 0.266667 |
| 72 | 0.24 |

**Table** **S5** Oviposition counts depicted as cumulative number of measurements per number of egg-rafts per predator treatment (left) and as spatial overview of the cumulative count (right). The numbers reflect the number of observations and therefore may include multiple mesocosms on the same day.

|  | Predator | | |
| --- | --- | --- | --- |
| Rafts | control | frog | newt |
| 0 | 118 | 110 | 105 |
| 1 | 18 | 0 | 5 |
| 2 | 3 | 0 | 0 |
| 3 | 1 | 0 | 0 |

**Table S6** Calculations average weight per predator species and corresponding maximum kill rates per gram of predator

| Species | Length (mm) | Weight (mg) | kill rate/gram | Reference length | | Reference weight | Formula |
| --- | --- | --- | --- | --- | --- | --- | --- |
| *A. bipustulatus* | 6.5-7.5 | 1.90-2.71 | 2215 | (Ohba & Takagi, 2010) | (Smock, 1980) | | 0.019*L^2.46 |
| *N. glauca* | 15-16 | 14.86-17.42 | 747 | (Reynaldi et al., 2011) | (Smock, 1980) | | 0.019*L^2.47 |
| *L. vulgaris* | 66.1-81.8 | 7070.97-13611.03 | 3 | (Bozkurt et al., 2016) | (Santini et al., 2018) | | 10^-4.375 * L^3.215 |
| *P. esculentus* | 35.3-48.6 | 22812.96-64797.45 | 1 | (Socha & Ogielska, 2010) | (Santini et al., 2018) | | 10^-4.744 * L^3.073 |

**References accompanying Table S6**

Bozkurt, E., Tural, M., Ulutaş, G., Üzüm, N., & Olgun, K. (2016). Two New Paedomorphic Population Records of the Smooth Newt, Lissotriton vulgaris schmidtleri (Raxworthy, 1988) (Urodela, Salamandridae), from Western Turkey. *Russian Journal of Herpetology*, *23*(2), Article 2. https://doi.org/10.30906/1026-2296-2016-23-2-158-162

Ohba, S.-Y., & Takagi, M. (2010). Predatory Ability of Adult Diving Beetles on the Japanese Encephalitis Vector Culex tritaeniorhynchus. *Journal of the American Mosquito Control Association*, *26*(1), 32–36. https://doi.org/10.2987/09-5946.1

Reynaldi, S., Meiser, M., & Liess, M. (2011). Effects of the pyrethroid fenvalerate on the alarm response and on the vulnerability of the mosquito larva *Culex pipiens molestus* to the predator *Notonecta glauca*. *Aquatic Toxicology*, *104*(1), 56–60. https://doi.org/10.1016/j.aquatox.2011.03.017

Santini, L., Benítez-López, A., Ficetola, G. F., & Huijbregts, M. A. J. (2018). Length–mass allometries in amphibians. *Integrative Zoology*, *13*(1), 36–45. https://doi.org/10.1111/1749-4877.12268

Smock, L. A. (1980). Relationships between body size and biomass of aquatic insects. *Freshwater Biology*, *10*(4), 375–383. https://doi.org/10.1111/j.1365-2427.1980.tb01211.x

Socha, M., & Ogielska, M. (2010). Age structure, size and growth rate of water frogs from central European natural Pelophylax ridibundus-Pelophylax esculentus mixed populations estimated by skeletochronology. *Amphibia-Reptilia*, *31*(2), 239–250. https://doi.org/10.1163/156853810791069119
